# The establishment of reference intervals for the ClotPro thromboelastometry device in healthy dogs

**DOI:** 10.1111/vec.13426

**Published:** 2024-11-18

**Authors:** Samantha K. Day, Katherine J. Nash, Mark J. Midwinter, Sarah L. Purcell, Wendy A. Goodwin

**Affiliations:** ^1^ School of Veterinary Science The University of Queensland Gatton Queensland Australia; ^2^ School of Biomedical Sciences The University of Queensland Gatton Queensland Australia

**Keywords:** coagulation, hemostasis, point of care, thromboelastometry, viscoelastic testing

## Abstract

**Objective:**

To establish reference intervals using a new point‐of‐care thromboelastometry device in dogs for the extrinsically activated test (EX‐test), intrinsically activated test (IN‐test), fibrin polymerization test (FIB‐test), ecarin test (ECA‐test), and tissue plasminogen activator test (TPA‐test) and to investigate the effects of storage time on the results.

**Design:**

Prospective clinical study in 2022.

**Setting:**

University teaching hospital.

**Animals:**

Forty‐eight healthy privately or university‐owned dogs were prospectively enrolled and included on the basis of normal physical examination and normal baseline laboratory results (CBC, biochemistry profile, prothrombin time, and activated partial thromboplastin time [aPTT]).

**Interventions:**

After a 30‐minute storage time, the EX‐test, IN‐test, FIB‐test, ECA‐test, and TPA‐test were performed on citrated blood samples. To determine the effect of storage time, 11 samples had the EX‐test, FIB‐test, and IN‐test repeated 90 and 150 minutes after sample collection.

**Measurements and Main Results:**

Ten thromboelastometry parameters were evaluated for each test. Reference intervals were calculated using the robust method for parametric data, and the robust Box–Cox transformed or nonparametric methods were used for nonparametric data. Increasing storage time resulted in more hypocoagulable tracings. A correlation was found between the IN‐test and aPTT (*r* = 0.62, *P* < 0.0001). Other weak to moderate correlations were seen between thromboelastometry parameters and platelet count and hematocrit.

**Conclusions and Clinical Importance:**

The development of reference intervals for the thromboelastometry device allows for the clinical use of this technology. Analyzing samples after a prolonged storage time of more than 30 minutes may result in erroneous results. Results may also be affected by an abnormal hematocrit or platelet count.

**Abbreviations**
A05: amplitude at 5 minutes
A10: amplitude at 10 minutes
A30: amplitude at 30 minutes
aPTT: activated thromboplastin time
CFT: clot formation time
CT: coagulation time
MCF: maximum clot firmness
ML: maximum lysis
PT: prothrombin time
ROTEM: rotational thromboelastometry
TEG: thromboelastography
TF: tissue factor

## 1 INTRODUCTION

Hemostatic disorders are common in critically ill patients and can prove rapidly fatal without prompt diagnosis and treatment. Traditional hemostatic tests such as the prothrombin time (PT) and activated partial thromboplastin time (aPTT) assess only specific aspects of plasma coagulation and do not provide insight into interactions between cellular surfaces and plasma clotting factors. Viscoelastic testing performed on whole blood has become a widely recognized method to evaluate hemostasis, allowing for all stages of initiation, amplification, and propagation to be assessed in a single, real‐time, near‐patient test that describes each stage of clot formation and dissolution.^1^, ^2^, ^3^, ^4^

A new‐generation point‐of‐care thromboelastometry device^a^ has been developed that uses an altered viscoelastometry measurement technique to assess global hemostasis. Although similar to well‐established viscoelastometry devices, the thromboelastometry device is smaller in size, suffers less environmental interference and variability, and allows remote viewing.^4^, ^5^ Compared with thromboelastography (TEG) and rotational thromboelastometry (ROTEM), in which the pin is the site of detection, a key difference in the thromboelastometry device is that the pin is held stationary while a rotational force is applied to the cylindrical cup, with detection and elastic coupling also linked to the cup. The viscoelastometry analyzer also uses “active tip” technology for the pipetting process, with reagent being preloaded in pipette tips, which ensures that samples are reconstituted with the correct concentration of reagent and enables a step that may otherwise allow the introduction of user error. Citrated whole blood is pipetted into the cup with an immersed pin and subsequently becomes activated. The blood adheres to the cup and pin surface, and the clot strength is continually measured and displayed graphically. Several reagents (active tips) are included with the analyzer that allow for the detection and assessment of primary and secondary hemostasis, factor deficiencies, fibrinogen, fibrinolysis, heparin and direct oral anticoagulant effects, and antifibrinolytic drug response (Table 1).^6^ Additionally, the device has 6 channels to facilitate multiple tests being performed in parallel.

**TABLE 1:** Viscoelastometric device test reagents.^5^, ^7^, ^8^, ^11^

Viscoelastometric test	Test description	Comparable ROTEM test
EX‐test	The extrinsic test provides a rapid overview of coagulation using CaCl_2_ for recalcification, polybrene as a heparin inhibitor, and TF as the activating agent.	EXTEM
IN‐test	The intrinsic screening test uses CaCl_2_ for recalcification and ellagic acid to initiate coagulation, and it is sensitive to heparin and coagulation factors such as FVIII.	INTEM
FIB‐test	A functional fibrinogen test that uses CaCl_2_ for recalcification, polybrene as a heparin inhibitor, TF for activation, and dual platelet inhibition with cytochalasin D and a glycoprotein IIb/IIIa inhibitor	FIBTEM
TPA‐test	A tissue plasminogen activator test to stimulate fibrinolysis	N/a
ECA‐test	A test containing polybrene and the prothrombin activator ecarin that allows detection and differentiation of antagonists to FXa and thrombin	N/a

Abbreviations: CaCl_2_, calcium chloride; FVIII, coagulation factor VIII; FXa, active coagulation factor X; N/a, not applicable; TF, tissue factor.

Despite being used in human health care and clinical research,^6^, ^7^, ^8^, ^9^, ^10^ there are currently no reports of the viscoelastometry device in veterinary medicine. In order to attain diagnostic accuracy, there is a need to establish species‐specific reference intervals. Additionally, viscoelastic testing is known to be influenced by a number of in vivo and in vitro factors, and evidence‐based guidelines are available for rotational viscoelastic testing, including recommendations for best practice in terms of sample storage times and methods, collection techniques, and the use of quality controls.^2^, ^3^, ^11^ Research investigating ROTEM and TEG in animals has found that samples tend to become more hypercoagulable with time, resulting in the recommendation that citrated whole blood be analyzed 30 minutes after collection. This allows for consistency in reading times between centers while providing time for transport and analysis of samples where required.^3^, ^12^, ^13^, ^14^, ^15^ Correlations between baseline variables, such as hematocrit and conventional coagulation tests, and TEG and ROTEM variables have been extensively studied to aid in appropriate interpretation of these viscoelastic tests.^12^, ^16^, ^17^, ^18^, ^19^, ^20^, ^21^ It is unknown whether these results can be extrapolated to this new analyzer because no studies have assessed the effect of storage time in human or animal samples.

The primary aim of the current study was to establish reference intervals for viscoelastometry parameters in healthy dogs. The secondary aim was to investigate the effect of storage time as well as evaluate the correlation of certain parameters with plasma coagulation tests, hematocrit, platelet count, and total protein. We hypothesized that increasing storage time would increase the hypercoagulability of the tracing.

## 2 MATERIALS AND METHODS

### 2.1 Study outline

This prospective observational study was conducted at a single veterinary hospital. The investigation was approved by the institutional Animal Ethics Committee (Approval number 2021/AE000818).

Forty‐eight healthy dogs were enrolled in the study. Animals were either privately owned or part of the institutional colony, and written informed owner consent was obtained before study inclusion. Dogs were eligible for inclusion if they were healthy based on results of a physical examination, were not currently receiving medication beyond routine prophylactic care (eg, flea, tick, intestinal worm, and heartworm preventatives), had not undergone blood sample collection in the previous 3 months, and had a body weight >3 kg. A CBC, biochemical profile, PT, and aPTT were performed; if values were >10% outside of the laboratory or device‐specific reference interval, dogs were excluded from the study. Dogs that became distressed during sample collection or that required more than 2 attempts at blood draw were also excluded.

### 2.2 Blood sampling

The area over the jugular vein was clipped and disinfected using a chlorhexidine‐alcohol swab. Blood samples were collected from the jugular vein with a 23‐Ga needle attached to a nonheparinized 10‐mL syringe. Blood was immediately transferred to a 2‐mL serum tube, a 2‐mL, 3.2% sodium citrate tube (ratio of 1 mL sodium citrate to 9 mL blood), and a 2‐mL EDTA tube (in that order), ensuring proper filling of collection tubes for the correct ratios of anticoagulant to blood. For 11 dogs, tubes were filled as described, but an additional 2‐mL, 3.2% sodium citrate tube was collected. Sodium citrate and EDTA tubes were immediately inverted at least 3 times to ensure adequate mixing. Serum and EDTA tubes were analyzed for CBC^b^ and biochemical analysis^c^. The 3.2% citrate tube had 0.2 mL removed to perform the PT and aPTT using a commercial analyzer^d^. The citrated samples were kept at room temperature (20–25°C) until viscoelastic testing was performed.

### 2.3 Viscoelastic analysis

All viscoelastic measurements were performed as per the Partnership on Rotational ViscoElastic Test Standardization (PROVETS) recommendations.^3^ To determine the device‐specific reference intervals, a single operator performed each of the following 5 tests in parallel 30 minutes after sample collection: extrinsically activated test (EX‐test), intrinsically activated test (IN‐test), fibrin polymerization test (FIB‐test), ecarin test (ECA‐test), and tissue plasminogen activator test (TPA‐test) (Table 1). To determine the effect of storage time, the additional 11 citrated samples were analyzed 90 and 150 minutes after collection for the EX‐test, IN‐test, and FIB‐test assays, as described.

All tests were performed according to the standard test protocol outlined in the viscoelastometric analyzer's user manual.^7^ Briefly, after entering patient details into the system, each cup and pin were loaded into the test positions on the 37°C prewarmed instrument bench. The appropriate test tip was loaded onto the electronic pipette, and 340 µL of citrated blood was transferred from the collection tube into the cup. To ensure adequate mixing, the blood was then redrawn into the pipette and replaced into the test cup. Connecting the pin to the cup initiated the test, which was run for 60 minutes, and parameters were automatically measured and graphically displayed by the viscoelastometric device (see Table 2 for description of parameters measured).

**TABLE 2:** Viscoelastic parameters measured by the viscoelastometric device.^5^

Parameter	Acronym	Units	Description
Coagulation time	CT	s	Time from test start until an amplitude of 2 mm is reached
Amplitude at 5 minutes	A05	mm	Amplitude (firmness) at 5 minutes
Amplitude at 10 minutes	A10	mm	Amplitude (firmness) at 10 minutes
Amplitude at 30 minutes	A30	mm	Amplitude (firmness) at 30 minutes
Maximal clot firmness	MCF	mm	Maximal amplitude reached during the test
Clot formation time	CFT	s	Time between 2 mm and 20 mm amplitude
α‐Angle	α	°	Angle between the baseline and a tangent to the clotting curve through the 2 mm point
Maximum lysis	ML	%	Maximum lysis detected during the run time described as a percentage difference between MCF and the lowest amplitude after MCF is reached
Clot lysis index at 30 minutes	CLI30	%	Clot lysis detected at 30 minutes, described as a percentage difference between MCF and the amplitude at 30 minutes
Clot lysis index at 45 minutes	CLI45	%	Clot lysis detected at 30 minutes, described as a percentage difference between MCF and the amplitude at 45 minutes

### 2.4 Statistical methods

Reference intervals were calculated following Friedrichs et al^22^ American Society for Veterinary Clinical Pathology veterinary species guidelines using a commercial spreadsheet^e^ and freely available add‐in.^23^ Normality was assessed with *Q*–*Q* plots, histograms, and the Anderson–Darling tests. For normal distributions, the robust method was used to calculate reference intervals. Robust Box–Cox transformed (symmetric data without outliers) or nonparametric (nonsymmetric or outliers) methods were used for nonnormal distributions.

Pearson correlation coefficients were estimated for the correlations of coagulation time (CT), aPTT, and PT. Because of the presence of outliers or nonnormal distributions, Spearman correlation coefficients were estimated for the correlations of CT, clot formation time (CFT), amplitude at 10 minutes (A10), α angle (α), and maximum clot firmness (MCF) with hematocrit, total plasma protein, and platelet count. Correlation analysis *P*‐values were adjusted with the linear step‐up false discovery rate method for multiple comparisons. Interpretation of the magnitude of the observed correlation coefficient was categorized as negligible to very strong, as described by Schober et al.^24^

For each parameter and reagent, linear mixed models were used to test for effects of storage times and viscoelastometric parameters. The models had a single fixed factor of time and a random intercept for each sample. Conditional model residuals and *Q*–*Q* plots were examined to confirm the assumption of normality. If normality was not confirmed, a Friedman test was used. Post hoc testing was performed using Dunnett's test for the linear mixed models and Conover tests for the Friedman tests. All analyses were performed using commercial software^f^. A significance threshold of 0.05 was used.

## 3 RESULTS

### 3.1 Population data

Of the 48 dogs enrolled in the study, 1 dog was excluded due to evidence of hepatopathy on screening bloodwork. Of the 47 dogs included in the study population, 25 were females (20 neutered, 5 entire) and 22 were male (17 neutered, 5 entire). The median (range) age was 4.75 years (4 months to 11 years). The median body weight was 21.0 kg (3.1–46.1 kg). Breeds of dogs included mixed breed (18), Miniature Dachshund (6), Border Collie (3), Labrador Retriever (3), Siberian Husky (3), Australian Cattle Dog (2), Jack Russell Terrier (2), Greyhound (2), and 1 each of Australian Kelpie, Australian Koolie, Rottweiler, English Springer Spaniel, German Shepherd Dog, Miniature Fox Terrier, Pomeranian, and Pug.

### 3.2 Reference intervals

Some individual tests were excluded from statistical analysis due to device errors during analysis (4), operator error in pipetting the sample (3), and reagent supply issues at the time of testing (4). Therefore, of the 47 included dogs, 44 samples were included in the statistical analysis of the EX‐test, 45 for the FIB‐test, 47 for the IN‐test and ECA‐test, and 41 for the TPA‐test.

Reference intervals and the upper and lower 90% CIs for each test parameter are reported in Table 3. Due to insufficient data, reference intervals were not calculated for the maximum lysis (ML) of the EX‐test, IN‐test, FIB‐test, and ECA‐test, CFT of the FIB‐test, ECA‐test, and TPA‐test, and A05, A10, and A30 of the TPA‐test.

**TABLE 3:** Reference intervals for viscoelastometric device parameters of the EX‐test, IN‐test, FIB‐test, ECA‐test, and TPA‐test in healthy dogs.

	Parameter (unit)	CT (s)	A05 (mm)	A10 (mm)	A30 (mm)	MCF (mm)	CFT (s)	ML (%)	CLI30 (%)	CLI45 (%)	Α (°)
EX‐test	*n*	45	45	45	45	45	45	15	45	45	45
	Reference interval	28.6–90.3	12–52.9	17.3–59	25.2–65.9	24.8–66.7	40.8–857.5	–	96.3–100	86.5–100	34.6–79.9
	90% CI LL	28–32.3	12–25	17–31	24–39.6	27–41.5	40–46.1	–	96–100	86–96.6	31–57.9
	90% CI UL	67.5–93	47–53	53–59	60.9–66	62.7–67	187–895	–	100–100	100–100	78.7–80
IN‐test	*n*	47^a^	47	47	47	47	46	16	47	47	47
	Reference interval	72.6–178.2	7.6–46.2	10.2–54.2	15.8–61.4	18–62.4	60.4–572.1	–	100–100	99–100	44.4–78.8
	90% CI LL	59.6–84.9	6–25.2	8–33	13–42.2	15–43.2	60–63	–	100–100	99–99	41–64.4
	90% CI UL	166.2–188.4	43–47	0.8–55	58.8–62	60–63	189.8–650	–	100–100	100–100	77.8–79
FIB‐test	*n*	44^a^	44	44	44	44	0	11	44	44	41
	Reference interval	14.8–68.1	3–12.9	3–13	3.1–13	3.1–13	–	–	90–100	88–100	16.2–80.9
	90% CI LL	10.1–19.3	3–3.1	3–4	3–4	3–4.1	–	–	90–92	88–90.4	15–42.1
	90% CI UL	61.8–75.7	11.9–13	12–13	12–13	12.9–13	–	–	100–100	100–100	76.9–81
ECA‐test	*n*	47	47	47	47	47	13	2	47	47	47
	Reference interval	251.8–735.4	3–7.8	3.2–16.6	6.2–45.6	7.2–52.8	–	–	90.8–100	95.6–100	4.2–25.8
	90% CI LL	247–372	3–3	3–5	6–7	7–9	–	–	89–99.2	95–100	4–6
	90% CI UL	704–738	6.8–8	10–18	20.8–51	29–58	–	–	100–100	100–100	19.6–26
TPA‐test	*n*	41	5	1	0	41	3	34^a^	41	41	39^b^
	Reference interval	18.2–65.5	–	–	–	3–28.6	–	80.7–95.7	7.2–72.6	7.2–72.9	57.4–80.9
	90% CI LL	18–22	–	–	–	3–6	–	79–82.6	7–10.1	7–11	52.1–62.2
	90% CI UL	51.3–66	–	–	–	20–29	–	94–97.4	39.7–73	39.6–73	79.6–82.2

Abbreviations: A05, amplitude at 5 minutes; A10, amplitude at 10 minutes; A30, amplitude at 30 minutes; CFT, clot formation time; CI LL, 90% CI for lower limit; CI UL, 90% CI for upper limit; CLI30, clot lysis at 30 minutes; CLI45, clot lysis at 45 minutes; CT, coagulation time; ECA‐test, ecarin test; EX‐test, extrinsically activated test; FIB‐test, fibrin polymerization test; IN‐test, intrinsically activated test; MCF, maximum clot firmness; ML, maximum lysis; *n*, number of data points included in value analysis; TPA‐test, tissue plasminogen activator test; α, α‐angle.

^a^ Robust Box–Cox untransformed method used for analysis.

^b^ Robust Box–Cox transformed method used for analysis.

### 3.3 Correlations between viscoelastometric parameters and plasma coagulation tests, hematocrit, platelet count, and total plasma protein

Correlations between PT, aPTT, hematocrit, and platelet count, and the FIB‐test, IN‐test, and EX‐test viscoelastometric parameters CT, A10, α‐angle, and MCF are shown in Table 4. Significant correlations were all classified as weak (*r* value = 0.10–0.39) or moderate (*r* value = 0.40–0.69).

**TABLE 4:** Correlations (*r*) between plasma coagulation times, hematocrit, total plasma protein concentration and platelet count, and viscoelastometric device parameters.

		EX‐test					IN‐test					FIB‐test			
Parameter		CT	A10	MCF	α	CFT	CT	A10	MCF	α	CFT	CT	A10	MCF	α
PT	*r* *P*	0.02 0.881	n/a	n/a	n/a	n/a	0.05 0.738	n/a	n/a	n/a	n/a	0.22 0.153	n/a	n/a	n/a
aPTT	*r* *P*	0.16 0.297	n/a	n/a	n/a	n/a	**0.62** **<0.001**	n/a	n/a	n/a	n/a	0.24 0.113	n/a	n/a	n/a
HCT	*r* *P*	0.19 0.219	**−0.31** **0.007**	**−0.39** **0.008**	**−0.41** **0.005**	**0.39** **0.009**	−0.15 0.30	**−0.50** **<0.001**	**−0.50** **<0.001**	**−0.47** **0.001**	**0.46** **0.001**	0.14 0.372	**−0.46** **0.002**	**−0.40** **0.006**	**−0.42** **0.006**
TPP	*r* *P*	−0.01 0.940	−0.19 0.219	−0.15 0.332	−0.16 0.289	0.16 0.302	0.14 0.341	−0.10 0.487	−0.10 0.485	−0.23 0.126	0.12 0.430	−0.22 0.149	−0.21 0.162	−0.21 0.178	−0.24 0.132
PLT	*r* *P*	**−0.33** **0.029**	**0.46** **0.001**	**0.39** **0.008**	**0.43** **0.003**	**−0.48** **0.001**	−0.05 0.718	**0.44** **0.002**	**0.41** **0.005**	**0.39** **0.007**	**−0.47** **0.001**	−0.09 0.569	−0.21 0.178	−0.01 0.940	−0.02 0.910

*Note*: Statistically significant correlations are in bold.

Abbreviations: A10, amplitude at 10 minutes; aPTT, activated partial thromboplastin time; CFT, clot formation time; CT, coagulation time; EX‐test, extrinsically activated test; FIB‐test, fibrin polymerization test; IN‐test, intrinsically activated test; MCF, maximum clot firmness; n/a, not analyzed; PLT, platelet count; PT, prothrombin time; TPP, total plasma protein; α, α‐angle.

### 3.4 The effect of sample storage time on viscoelastometric parameters

Descriptive results for the viscoelastometric parameters for the EX‐test, IN‐test, and FIB‐test analyzed 30, 90 and 150 minutes after collection are shown in Table 5. For all 3 tests, values reflecting clot firmness (A05, A10, A30, and MCF) trended lower over time for the EX‐test and IN‐test, with CFT values trending higher over time.

**TABLE 5:** Mean values (with 2 SDs from the mean range) of the viscoelastometric device parameters analyzed 30, 90, and 150 minutes after collection in 11 dogs.

Test	Time (min)	CT (s)^a^	A05 (mm)	A10 (mm)	A30 (mm)	MCF (mm)	CFT (s)^a^	ML (%)^a^, ^b^	CLI30 (%)^a^	CLI45 (%)^a^	α (°)
EX‐test	30	45 (39–50)	33.91 (22.31–45.51)	41.82 (30.3–53.34)	50.73 (40.69–60.77)	52.73 (43.87–61.59)	112 (85–135)	2 (2–2)	100 (100–100)	100 (100–100)	69.82 (60.36–79.28)
	90	48 (43–53)	31.82 (15.52–48.12)	39.73 (22.36–57.09)	48.82 (30.98–66.66)	52.27 (31.11–73.43)	127 (77–155)	2 (1–3)	100 (100–100)	100 (100–100)	65.55 (46.85–84.25)
	150	46 (41–50)	28.73 (11.41–40.05)	37 (17.7–56.3)	46.82 (27.22–66.42)	48.45 (29.17–67.73)	165 (92–197)	1.5 (1–2)	100 (100–100)	100 (100–100)	60.91 (29.29–92.53)
IN‐test	30	134 (96–140)	32.18 (22.1–42.26)	40.36 (29.36–51.36)	50.36 (39.84–60.88)	52.27 (42.55–61.99)	125 (92–150)	1.5 (1–2.5)	100 (100–100)	100 (100–100)	70.82 (63.08–78.56)
	90	139 (132–153)	30 (15.38–44.62)	37.91 (21.75–54.07)	47.73 (31.57–63.89)	49.45 (34.51–64.39)	137 (100–200)	2 (1–3)	100 (100–100)	100 (100–100)	66.64 (53.24–80.04)
	150	135 (127–149)	29.18 (13.04–45.32	36.82 (19.32–54.32)	46.36 (29.02–63.7)	48.09 (31.01–65.17)	130 (95–230)	2 (1–3)	100 (100–100)	100 (100–100)	64.64 (42.14–87.14)
FIB‐test	30	44 (33–46)	6.91 (0.63–13.19)	7.27 (1.33–13.21)	7.36 (1.34–13.38)	7.36 (1.34–13.38)		1 (1–2)	99 (97–100)	98 (96–99)	57.45 (35.61–79.29)
	90	42 (34–49)	6.36 (0.22–12.5)	6.73 (0.27–13.19)	6.73 (0.53–12.93)	7.18 (1.12–13.24)		8.5 (2–15)	98 (95–100)	98 (94–99)	50.27 (0–102.77)
	150	44 (34–59)	5.82 (0.94–10.7)	6.09 (0.91–11.27)	6.09 (1.23–10.95)	6.36 (1.68–11.04)		5 (5–5)	99 (96–99)	98 (97–99)	46.73 (0–94.43)

*Note*: No results are reported for the FIB‐test CFT given the amplitude did not reach the 20 mm required to record a CFT.

Abbreviations: A05, amplitude at 5 minutes; A10, amplitude at 10 minutes; A30, amplitude at 30 minutes; aPTT, activated partial thromboplastin time; CFT, clot formation time; CI LL, 90% CI for lower limit; CI UL, 90% CI for upper limit; CLI30, clot lysis at 30 minutes; CLI45, clot lysis at 45 minutes; CT, coagulation time; EX‐test, extrinsically activated test; FIB‐test, fibrin polymerization test; IN‐test, intrinsically activated test; ML, maximum lysis; α, α‐angle.

^a^ Nonnormally distributed data reported as median with lower and upper quartile range in brackets.

^b^ Less than total 11 data points were available for analysis, due to a value not being generated due to no lysis being detected by completion of the test.

Paired comparisons found statistically significant differences when comparing the values at 30 versus 90 minutes for the following parameters of the EX‐test (A05: *P *= 0.002; A10: *P *= 0.009; A30: *P *= 0.037; CFT: *P *= 0.002; α‐angle: *P *= 0.016), at 30 versus 90 minutes for the IN‐test (CT: *P *= 0.026), and at 30 versus 150 minutes for the IN‐test (CT: *P *= 0.026; A30: *P *= 0.039; MCF: *P *= 0.048; CFT: *P *= 0.012; α‐angle: *P *= 0.036). Values reflecting clot firmness (A05, A10, A30, and MCF) trended lower over time, with CFT values trending higher over time, thus reflecting a more hypocoagulable state as storage time increased. This trend was also observed in the FIB‐test values over increasing time, but statistical significance was not reached.

## 4 DISCUSSION

This is the first study to report reference intervals for the viscoelastometric analyzer in dogs. The development of species‐specific reference intervals is imperative. Viscoelastic reference intervals specific to people cannot be extrapolated to dogs due to species‐specific differences in fibrinogen concentration, thrombin formation, fibrinolysis, and other contributing factors that affect clot formation, strength, and dissolution.^25^

The more traditional tests offered by the analyzer in this study include the EX‐test, IN‐test, and FIB‐test, which are similar to the ROTEM EXTEM, INTEM, and FIBTEM, respectively. Although similar parameters are reported, the assays are not identical, with small differences in the assay contents and device mechanics.^26^ The use of tissue factor (TF) and polybrene with calcium chloride for recalcification of the citrated sample provides a rapid overview of hemostasis observed with the EX‐test and is useful for a baseline assessment of coagulation status with consideration of the contributions of platelets and fibrinolytic factors in any patient to guide diagnosis and direct treatment. The IN‐test differs in that it uses ellagic acid to initiate coagulation without a heparin inhibitor, allowing a sensitivity to heparin and coagulation factors such as FVIII. The FIB‐test, similar to the EX‐test, uses TF and polybrene with calcium chloride for recalcification; however, the test also differs because it uses cytochalasin D and glycoprotein IIb/IIIa inhibitor for dual platelet inhibition to allow the assessment of functional fibrinogen in a sample.

Given that viscoelastometric and other ROTEM devices have many methodological similarities, it is of clinical interest to know whether the results of these analyzers can be used interchangeably. In people with COVID‐19 or undergoing cardiac bypass, paired measurements using the viscoelastometric and the thromboelastometry^g^ analyzers found strong correlations between many EX‐test, IN‐test, and FIB‐test parameters and their corresponding EXTEM, INTEM, and FIBTEM parameters.^7^, ^9^ However, both studies demonstrated multiple values that were either not correlated or only weakly to moderately correlated, thus highlighting the need for device‐specific reference intervals. The reference intervals reported for ROTEM in dogs had most, but not all, median values for each parameter fall within the reference intervals reported for the viscoelastometric device in the current study.^11^, ^12^ Results cannot be directly compared due to different populations, institutions, and methods, but this provides further evidence that reference intervals between these devices are not interchangeable. Furthermore, increasing storage time in this study resulted in reductions in clot firmness and increased CFT, and thus the reference intervals reported should only be applied to samples run at similarly collected samples stored for 30 minutes.

Also assessed in this study were the viscoelastometric ECA‐test and TPA‐test. The ECA‐test uses ecarin, isolated from viper venom (*Echis carinatus*), to activate prothrombin, form meizothrombin, and thus cleave fibrinogen to fibrin, detected by the CT. As the test reagent also contains polybrene, heparin is antagonized if present. As a result, this test is more sensitive to fibrinolysis and detects it much sooner than the traditional tests, which is important in critically unwell or at‐risk patients, such as those undergoing major surgery. In veterinary medicine, the test may be able to identify breeds at increased risk of hyperfibrinolysis.^27^, ^28^ It has been designed to allow detection and differentiation of antagonists to FXa and thrombin, but this is yet to be elucidated in animals. The TPA‐test complements the ECA‐test by using recombinant tissue plasminogen activator to stimulate fibrinolysis. It becomes clinically useful in demonstrating the failure of clot resolution by indicating antifibrinolysis and is hypothesized to be also useful in assessing the response to antifibrinolytic medications such as tranexamic acid and ε‐aminocaproic acid.^29^, ^30^ Using these newer tests could prove instrumental in determining the appropriate doses and efficacy of these medications in dogs and cats.

In the current study, multiple tests had no values recorded for certain parameters. For example, no values were recorded for the FIB‐test CFT as the test never reached the amplitude of 20 mm required for generation of this value. This was likely due to the weaker clot firmness attained in the face of platelet inhibition, consistent with studies in healthy and sick human patients in which CFT is not used for this reason.^7^ The lack of CFT in the ECA‐test and TPA‐test was also the result of failure to reach an amplitude of 20 mm in these tests given the rapid fibrinolysis in most samples. It is possible that reference intervals for these values could have been generated with more samples. The parameters of A05, A10, and A30 in the TPA‐test failed to record values in most samples because the small clot that was formed had undergone sufficient fibrinolysis by these time points; therefore, no amplitude was registered. ML was not reported for the EX‐test, IN‐test, FIB‐test, and ECA‐test because the MCF amplitude achieved did not decrease during the test run time and calculation of an ML in most cases was not allowed. Therefore, appropriate statistical analysis could not be performed because insufficient values were attained for each of these tests.

In this study population of healthy dogs, the parameters of the viscoelastometric device were significantly correlated with changes in patient PT, hematocrit, and platelet count. For example, a moderate correlation was observed between the IN‐test CT and aPTT. The CT parameter of the device generally reflects the initiation phase of coagulation, and each test is impacted by the activating agent used as well as by the available factor concentration for the generation of thrombin to prime the amplification and propagation phases.^13^, ^31^, ^32^ The PT and aPTT tests use activators similar to those used to initiate the EX‐test and IN‐test, respectively; however, the former tests lack the cellular surfaces that allow viscoelastic tests such as the analyzer in the current study to more accurately reflect in vivo hemostasis.^16^ The IN‐test CT of the viscoelastometric device has also been shown to have a strong correlation with aPTT in people with COVID‐19 and in people undergoing cardiac surgery.^7^, ^9^ Similarly, a weak to moderate correlation has been shown between the ROTEM INTEM CT and aPTT in people.^7^, ^17^ Although Enk et al^18^ found a strong correlation between ROTEM INTEM CT and aPPT in dogs, other studies failed to find a correlation.^12^, ^16^ Interestingly, in the same study of people with COVID‐19, a much weaker correlation between ROTEM INTEM CT and PT was reported than between the IN‐test CT and PT of the viscoelastometric device.^7^ This again highlights that while similarities exist between tests and parameters, results, including correlations, are not interchangeable. This comparison is supported by the positive correlation between the IN‐test and PT of the viscoelastometric device in our study, which has not previously been present in studies investigating ROTEM INTEM CT and PT in dogs. The lack of correlation between PT and EX‐test CT in our study contrasts the findings of a moderate to strong correlation observed between PT and ROTEM EXTEM in both healthy and sick dogs.^12^, ^16^, ^18^ In people, correlations between EX‐test CT and PT have also been varied, with some showing a correlation and others not.^7^, ^9^

Hematocrit was moderately correlated with the EX‐test α and weakly correlated with the EX‐test A10, MCF, and CFT. Hematocrit also showed a significant moderate correlation with the IN‐test A10, MCF, α, and CFT and with the FIB‐test A10, MCF, and α. Generally, values of clot firmness showed an inverse relationship in that a lower hematocrit was associated with increased clot firmness and thus trended toward hypercoagulability. A trend toward hypercoagulability was reflected by a proportional correlation in CFT, indicating a faster clot formation with lower hematocrit. It is important to note that all hematocrit values in this study were within the normal reference interval as per the study design; thus, findings do not reflect or predict correlations in dogs with abnormally low or high hematocrits. Further investigation is needed to see if this correlation results in clinically relevant findings. Hypercoagulability with decreased red blood cell mass has been repeatably shown in dogs.^16^, ^19^, ^20^ It has previously been hypothesized that the hypercoagulability may be due to a larger plasma fraction; however, this does not align with the clinical scenario where anemic patients typically have prolonged bleeding times. The theory that viscosity drives hypercoagulable thromboelastographic tracings was supported by the demonstration of hypocoagulable tracings in anemic samples that had been normalized for viscosity.^33^ This emphasizes the need to use caution when interpreting any thromboelastometry result in dogs with abnormal hematocrits.

Platelet count showed a moderate correlation with the EX‐test A10, α, and CFT and with the IN‐test A10, MCF, α, and CFT, with values reflecting clot firmness (A10, MCF, α) positively correlated and CFT negatively correlated with platelet count. This is not unexpected and is supported by similar thromboelastic studies that investigated the effect of platelet count.^7^, ^16^, ^17^, ^21^ It is highly likely that stronger correlations will be identified in dogs with abnormalities such as thrombocytopenia or thrombocytopathia, anemia, or polycythemia.

With regard to benchtop storage times after blood collection, PROVETS guidelines recommend a 30‐minute storage time for citrate anticoagulated blood samples when establishing species‐specific reference intervals.^2^, ^3^, ^11^, ^34^ However, performing an analysis of a sample 30 minutes after collection is not always feasible. In some instances, this is due to the need for prompt results in an unstable patient; in others, it may result from delays in running tests due to clinical duties, lack of available trained device operators, or location or sample transport requirements. No studies have assessed the effect of variable sample storage time on the viscoelastometric device parameters in people or animals. Studies in people have run samples between 0 and 40 minutes after collection but have not specifically examined the effect of storage time.^7^, ^9^ Increasing storage time in the current study led to decreased clot firmness and increased CFT; therefore, the reference intervals reported should only be applied to samples run at similarly collected samples stored for 30 minutes.

In studies of healthy people using similar devices, no influence of storage times of 0–120 minutes on ROTEM parameters was identified.^35^ In contrast, a study using TEG identified a trend toward hypercoagulability in citrated blood samples run between 30 and 60 minutes after collection, whereas minimal changes in results were seen in samples run between 1 and 8 hours, and another demonstrated hypercoagulability between 30 minutes and 4 hours with a period of stability between 30 minutes and 2 hours.^36^, ^37^ When assessing coagulopathy in trauma patients using ROTEM, significant changes were found over 0–60 minutes, with improvement of hypocoagulable tracings over time.^38^

In veterinary research, limited studies identified a similar trend in both TEG and ROTEM analysis toward hypercoagulability with increasing storage time, albeit the results were not always consistent.^13^, ^14^, ^15^ Most recently, a study using another thromboelastometry analyzer^h^ in 71 dogs had similar findings to our study, with a trend toward hypocoagulability over 70 minutes.^39^ These results were clinically relevant as samples that were hypocoagulable at 30 minutes were considered normocoagulable using the same reference interval at 10 minutes. Weingand et al^39^ were the first to report a trend toward hypocoagulability over time, suggesting this could result from initial platelet activation and aggregation associated with sampling that resolved over time. In the current study, this difference could hypothetically lead to a clinically relevant misinterpretation of a normocoagulable dog as hypocoagulable; however, given the small sample size used for this portion of our study and the inclusion of only healthy patients, the effect of storage time requires further investigation.

The greatest limitation of this study was that despite the inclusion of more than 40 individuals as per the American Society for Veterinary Clinical Pathology guidelines, nonparametric methods were required for the development of reference intervals of multiple parameters; thus, extrapolation of these results to the general population should be performed with caution. A larger sample population would likely have allowed parametric methods to be applied to all values and may have reduced any bias of convenience sampling that may have occurred despite precautions taken by adhering to inclusion and exclusion criteria. Furthermore, blood samples were not tested in duplicate because the viscoelastometric device in the current study has 6 channels, and the 5 different tests were performed simultaneously on each sample to adhere to standard storage times. Although some studies suggest duplicate assays are not required when using a ROTEM machine, others suggest a small coefficient of variation when duplicates are tested, which could influence results.^12^, ^40^ Some individual test values were excluded due to device or operator error. These errors could be further minimized with increased operator training and consideration of performing duplicate assays.

It is well documented that Greyhounds have hemostatic parameters that differ from other breeds, and although 2 Greyhounds were included in this study population, their individual results fell well within the established reference intervals. The influence of individual breed and age has not been considered and could influence results, especially when considering the increased incidence of conditions affecting hemostasis in certain breeds.^27^

In conclusion, the reporting of standard reference intervals using the viscoelastometric device in dogs allows clinical use of this technology to assess hemostasis in a variety of case presentations, while taking into consideration the methods of sample storage. Correlations of the device's parameters with aPTT, hematocrit, and platelet count should be considered when interpreting results.

## AUTHOR CONTRIBUTIONS

**Samantha K. Day**: Conceptualization; data curation; formal analysis; funding acquisition; investigation; methodology; project administration; validation; writing—original draft; writing—review and editing. **Katherine J. Nash**: Conceptualization; data curation; funding acquisition; investigation; methodology; supervision; validation; writing—review and editing. **Mark J. Midwinter**: Conceptualization; methodology; resources; supervision; writing—review and editing. **Sarah L. Purcell**: Conceptualization; supervision; writing—review and editing. **Wendy A. Goodwin**: Conceptualization; data curation; funding acquisition; methodology; project administration; resources; supervision; validation; writing—review and editing.

## CONFLICT OF INTEREST STATEMENT

The authors declare no conflicts of interest.

## ENDNOTES

a ClotPro, Hemonetics Corporation, Boston, MA.

b Sysmex XN‐1000 Hematology Analyzer, Sysmex Corporation, Kobe, Japan.

c AU480 Chemistry Analyzer, Beckman Coulter Ireland Inc., Co., Clare, Ireland.

d IDEXX Coag Dx Analyzer, IDEXX Laboratories, Westbrook, ME.

e Microsoft Excel, Microsoft, Redmond, WA.

f SAS 9.4, SAS Institute, Cary, NC.

g ROTEM Delta, Tem Innovations GmbH, Munich, Germany.

h ROTEM S, Tem Innovations GmbH, Munich, Germany.
